# Generation of Virus- and dsRNA-Derived siRNAs with Species-Dependent Length in Insects

**DOI:** 10.3390/v11080738

**Published:** 2019-08-11

**Authors:** Dulce Santos, Lina Mingels, Elise Vogel, Luoluo Wang, Olivier Christiaens, Kaat Cappelle, Niels Wynant, Yannick Gansemans, Filip Van Nieuwerburgh, Guy Smagghe, Luc Swevers, Jozef Vanden Broeck

**Affiliations:** 1 Research Group of Molecular Developmental Physiology and Signal Transduction, KU Leuven, 3000 Leuven, Belgium; 2 Laboratory of Agrozoology, Department of Plants and Crops, Faculty of Bioscience Engineering, Ghent University, 9000 Ghent, Belgium; 3 Laboratory of Pharmaceutical Biotechnology, Faculty of Pharmaceutical Sciences, Ghent University, 9000 Ghent, Belgium; 4 Insect Molecular Genetics and Biotechnology Group, Institute of Biosciences and Applications, National Center for Scientific Research “Demokritos”, 15310 Athens, Greece

**Keywords:** insects, RNA interference, small interfering RNAs, small interfering RNA length, dicer, pest control, viruses, CrPV, FHV, dsRNA

## Abstract

Double-stranded RNA (dsRNA) molecules of viral origin trigger a post-transcriptional gene-silencing mechanism called RNA interference (RNAi). Specifically, virally derived dsRNA is recognized and cleaved by the enzyme Dicer2 into short interfering RNAs (siRNAs), which further direct sequence-specific RNA silencing, ultimately silencing replication of the virus. Notably, RNAi can also be artificially triggered by the delivery of gene-specific dsRNA, thereby leading to endogenous gene silencing. This is a widely used technology that holds great potential to contribute to novel pest control strategies. In this regard, research efforts have been set to find methods to efficiently trigger RNAi in the field. In this article, we demonstrate the generation of dsRNA- and/or virus-derived siRNAs—the main RNAi effectors—in six insect species belonging to five economically important orders (Lepidoptera, Orthoptera, Hymenoptera, Coleoptera, and Diptera). In addition, we describe that the siRNA length distribution is species-dependent. Taken together, our results reveal interspecies variability in the (antiviral) RNAi mechanism in insects and show promise to contribute to future research on (viral-based) RNAi-triggering mechanisms in this class of animals.

## 1. Introduction

RNA interference (RNAi) is a post-transcriptional gene-silencing mechanism triggered by double-stranded RNA (dsRNA) molecules. As these RNA duplexes are mainly produced during the replicative cycle of viruses, RNAi is an important antiviral response. In insects, this pathway can be described in three central steps. First, the trigger dsRNA is recognized by the RNase III enzyme Dicer2 and processed into small RNA duplexes called small interfering RNAs (siRNAs). Second, these siRNAs (18–24 nt long) are loaded into the RNA-induced silencing complex (RISC) and unwound. The RISC then uses the so-called guide strand as a template to find the target transcript by Watson–Crick base pairing. In a third step, the guide strand directs the RISC endonuclease Argonaute2 to degrade the target transcript, thereby combating the viral infection. In this way, siRNAs determine the sequence specificity of the RNAi mechanism [1]. In spite of the vast diversity of the class Insecta, studies regarding siRNA structural features have been mostly carried out in the model organism *Drosophila melanogaster* [2,3,4].

DsRNA is an important pathogen-associated molecular pattern (PAMP) for viral infection, triggering a robust degradation of viral RNA via RNAi. In the case of RNA viruses, the presence of viral dsRNA during infection is very intuitive. For instance, during the replication cycle, the single-stranded (ss)RNA genome serves as a template for the production of complementary RNAs through the viral polymerase, forming a temporary dsRNA structure. In other cases, the viral genome directly consists of dsRNA molecules. Regarding viruses with a DNA genome, it is so far unclear which mechanisms lead to dsRNA occurrence. Nevertheless, it has been proposed that the presence of secondary structures in the viral transcript or the formation of complementary RNA sequences during the viral replication could be potential sources [1,5,6].

While RNAi is naturally activated as an antiviral defense mechanism, it can also be triggered by artificial delivery of gene-specific long dsRNA, resulting in specific endogenous gene silencing. This is called RNAi technology, a diverse technique that finds many applications both for research and commercial purposes. For example, RNAi-based approaches have been proposed to protect beneficial insects from harmful viral infections [7]. Moreover, RNAi technology is widely used as a loss-of-function research tool, which is particularly important for reverse genetic studies in nonmodel organisms. Most importantly, however, RNAi technology holds great promise to contribute to novel strategies for selectively controlling agricultural insect pests. In fact, an RNAi-based pesticide has already been approved by the United States Environmental Protection Agency (EPA) [8].

A general awareness regarding the use of so-called traditional pesticides is increasing worldwide. The main concerns consist of their lack of specificity, posing a threat to human health and contributing to a decrease in biodiversity. Notably, additional collateral damage to ecosystems can be evoked due to the development of resistance in insect populations. Insect resistance highly compromises pesticide efficiency, thus threatening food production. In this regard, the potential use of alternative options, such as RNAi technology, gains a new foothold. Although RNAi technology works efficiently in several insects, this is not the case for every species. Multiple factors are known to contribute to these difficulties, with the lack of an efficient dsRNA uptake mechanism by the insect cells being one of the crucial limiting steps. Therefore, bearing in mind the pest control potential of RNAi, research efforts are being set to obtain effective dsRNA delivery systems in insects. In this regard, viruses could be an interesting tool, mainly via three distinct promising approaches. The first one would consist of direct dsRNA delivery in virus(-like) particles, for instance originating from viruses with a dsRNA genome. A second possibility comprises of engineering the genome of a ssRNA virus to contain the selected RNA sequence. In a third approach, a DNA virus could be used, containing inverted repeats of the selected sequence. In the latter two, viral replication would then ensure intracellular dsRNA formation [7,9]. A recent successful example using the Flock House virus (FHV), a positive single-stranded (+ss)RNA virus with a bipartite genome (Nodaviridae family), provided proof of concept for the use of viruses to trigger RNAi in pest control [10]. These positive results encourage the search for other viral-based RNAi-triggering systems. On this, the Cricket Paralysis Virus (CrPV) is a potential candidate. CrPV (Dicistroviridae family) is a well-known insect virus whose genome is formed by one single molecule of +ssRNA. Although FHV and CrPV are well-known insect viral models, their host immune response is mainly described in the fruit fly *Drosophila melanogaster* and in its cultured cell lines, where it is often used to investigate RNAi-based antiviral immunity.

It is evident that the interspecies differences in the RNAi mechanism exist and remain largely unresolved, which strongly reflects on the practical applications of insect-related RNAi technology [7,9,11]. Therefore, following the knowledge obtained in the model organism *D. melanogaster*, research on a diverse number of other insect species is becoming more and more relevant. 

In this article, we start by reporting the generation of CrPV-derived siRNAs with species-dependent length distribution in five economically important insects, either in vivo or in vitro. Then, by using distinct viruses (the FHV, the Macula-like Latent Virus, the *Drosophila* A Virus, and the *Drosophila* C Virus), we demonstrate that the species-dependent siRNA length is not virus-specific. In addition, we show that siRNAs derived from dsRNA molecules also exhibit such a feature. Taken together, we describe the species-dependent length distribution of dsRNA- and/or virus-derived siRNAs in six insect species belonging to five orders: *Spodoptera exigua* and *Trichoplusia ni* (Lepidoptera); *Locusta migratoria* (Orthoptera); *Bombus terrestris* (Hymenoptera); *Tribolium castaneum* (Coleoptera); and *Drosophila melanogaster* (Diptera). Our results contribute to the understanding of interspecies variability of the (antiviral) RNAi mechanism in the class Insecta, as well as promise to contribute to future research on insect (viral-based) RNAi-triggering mechanisms.

## 2. Material and Methods

### 2.1. Insect Rearing

Beet armyworms and migratory locusts were available to the labs participating in this study, while bumblebees were obtained from a commercial supplier.

Beet armyworms (*S. exigua*) were reared under crowded conditions at 25 °C with a 16:8 h light/dark period and 65% relative humidity. During larval development, the caterpillars were continuously fed with an artificial diet, as described previously [12]. Adult moths were kept in large cages for mating and ovipositing and were fed with a 1:6 honey/water solution. 

Locusts (*L. migratoria*) were reared under crowded conditions at 32 °C ± 1 °C, with a 14:10 h light/dark period and 40%–60% relative humidity. Locusts were fed daily with grass, supplemented with dry oat flakes. Adult locusts were developmentally synchronized on the day of the last molt. 

Bumblebee (*B. terrestris*) callow workers were obtained from Biobest (Westerlo, Belgium). The callow or newborn workers were randomly collected from small queen-right colonies at their initial phase of start-up. Upon arrival in the lab, bumblebees were randomly transferred into different plastic microcolonies (20 bees/colony). These microcolonies were placed in an incubator at 30 °C, with 60% relative humidity, and in continuous darkness [13]. The bees were fed with gamma-irradiated pollen (Apihurdes, Pinofranqueado, Spain) and a water-based sugar solution (50%, *v*/*w*, BIOGLUC, Biobest).

### 2.2. Cell Culture

The selected cell lines were available to the labs participating in this study.

High Five cells (*Trichoplusia ni*) were maintained at 27.5 °C in IPL-41 Insect Medium (Sigma-Aldrich, Bornem, Belgium), supplemented with 10% heat-inactivated fetal bovine serum (FBS) (Sigma-Aldrich), 0.25 µg/mL amphotericin B (Sigma-Aldrich), 100 U/mL penicillin, and 100 µg/mL streptomycin (Gibco, Life Technologies, Merelbeke, Belgium). 

TcA cells (*Tribolium castaneum*) were maintained at 27.5 °C in EX-CELL^®^ 420 medium (Sigma-Aldrich), supplemented with 10% heat-inactivated FBS (Sigma-Aldrich), 5 µg/mL human insulin (Sigma-Aldrich), 0.25 µg/mL amphotericin B (Sigma-Aldrich), 100 U/mL penicillin, and 100 µg/mL streptomycin (Gibco, Life Technologies, Merelbeke, Belgium). 

S2 cells (*D. melanogaster*) were maintained at 25 °C in Shields and Sang M3 Insect Medium (Sigma-Aldrich), supplemented with 1 g/L yeast extract (Sigma-Aldrich), 2.5 g/L Bacto™ Peptone (BD Biosciences, San Jose, CA, USA), 10% heat-inactivated FBS (Sigma-Aldrich), 0.25 µg/mL amphotericin B (Sigma-Aldrich), 100 U/mL penicillin, and 100 µg/mL streptomycin (Gibco, Life Technologies).

### 2.3. Viral Production and Quantification

The CrPV and FHV suspensions were produced in cultured *D. melanogaster* S2 cells as previously described [14] and were concentrated by ultracentrifugation. The final viral pellet was resuspended in phosphate-buffered saline (PBS) (Sigma-Aldrich). The viral concentration was determined by negative staining, followed by transmission electron microscopy by CODA-CERVA (Ukkel, Belgium).

### 2.4. Viral Infections and Sample Collection

Five male adult *S. exigua* (2 days after emergence) were immobilized at 4 °C for 15 min and were intra-abdominally injected with CrPV suspension (1.5 × 10^7^ CrPV particles per animal). On day 3 postinjection, fat body was dissected in PBS under a binocular microscope and immediately transferred to liquid nitrogen. These samples were pooled and stored at −80 °C until further processing.

Six adult male *L. migratoria* were intra-abdominally injected with either CrPV or FHV viral suspension (10^7^ CrPV particles per animal; 10^9^ FHV particles per animal). On day 3 and day 5 postinjection, fat body was dissected in PBS under a binocular microscope and immediately transferred to liquid nitrogen. These samples were pooled and stored at −80 °C until further processing.

Five three-day-old *B. terrestris* workers were immobilized on ice for 2 min and were intra-abdominally injected with CrPV suspension (5 × 10^6^ CrPV particles per animal). On day 3 postinjection, fat body was dissected in PBS under a binocular microscope and immediately transferred to liquid nitrogen. These samples were pooled and stored at −80 °C until further processing.

High Five cells were collected by centrifugation, resuspended in the CrPV suspension with a dose of 25 viral particles per cell, diluted in IPL-41 Insect Medium (Sigma-Aldrich), and incubated at room temperature on a shaker plate for 2  h. Then, following a washing step in IPL-41 Insect Medium (Sigma-Aldrich), the cells were resuspended in complete medium (see “Cell Culture” section), plated in 24-well plates, and maintained at 27.5 °C. On day 2 postinfection, the cells were harvested by means of a scraper and collected for further analysis. Samples were stored at −80 °C until further processing.

TcA cells were plated at around 80% confluency and were grown in complete medium (see “Cell Culture” section) for 3 days at 27.5 °C. Then, following a washing step in EX-CELL 420 medium (Sigma-Aldrich), the cells were resuspended in EX-CELL 420 medium (Sigma-Aldrich), infected with 5 × 10^7^ viral particles of either FHV or CrPV, and maintained at 27.5 °C. On day 3 postinfection, the cells were harvested by means of a scraper and collected for further analysis. Samples were stored at −80 °C until further processing.

### 2.5. dsRNA Treatment and Sample Collection

Double-stranded RNA for *luciferase* (ds*luc*) (1054 nt) was kindly provided by Syngenta Ghent, Belgium.

90%–95% confluent S2 cells were incubated with ds*luc* (8 µg/mL) for 2 h, after which the cells were washed with PBS and incubated overnight in Shields and Sang M3 Insect Medium (Sigma-Aldrich). Next, the cells were harvested by means of a scraper and collected for further analysis. Samples were stored at −80 °C until further processing.

One adult male and one female *L. migratoria* were intra-abdominally injected with 500 ng ds*luc*. At 24 h postinjection, the midguts were dissected in PBS under a binocular microscope and immediately transferred to liquid nitrogen. These samples were pooled and stored at −80 °C until further processing.

### 2.6. RNA Extraction

Small (s)RNA extractions were performed with the miRNeasy Mini kit (Qiagen, Hilden, Germany) according to the manufacturer’s protocols. Quality and concentration of the extracted sRNA molecules were assessed with the 2100 Bioanalyzer Small RNA kit (Agilent Technologies, Leuven, Belgium). Samples were pooled prior to sequencing and stored at −80 °C until further processing.

### 2.7. Small RNA Sequencing and Data Analysis

The sRNA library preparations and next-generation sequencing were performed by NXTGNT (Gent). Briefly, sequencing libraries were prepared with the TailorMix microRNA Sample Preparation Kit V2 (SeqMatic, Fremont, USA) according to the manufacturer’s protocol. Next, these were sequenced in the Illumina MiSeq System, with a single-read length of 50 nt. PhiX spike-ins (2%) were added to monitor sequencing quality. Adapters were removed from the raw reads with BBDuk, version 37.33 (part of BBMap suite) [15]. FastQC (version 0.11.8) [16] was used to check the quality and length distribution of the reads.

In order to identify persistently infecting viruses, the libraries were analyzed based on previously reported methodology [17,18]. Briefly, in order to assemble the sRNAs into contigs, the Velvet program (version 1.2.10) [19] was used with a K-mer value of 17. The obtained contigs were used to perform a BLASTn search against all publicly available viral genome sequences (downloaded from NCBI) using the Blast2GO program [18] (version 5.2) [20].

In order to map the sRNA reads to the viral and dsRNA sequences, Bowtie2 (version 2.1.0) [21] was used, applying the sensitive preset parameters as described in the corresponding manual. The resulting Sequence Alignment/Map (SAM) files were converted to Binary Alignment/Map (BAM) format and were indexed and sorted using SAMtools (version 1.6) [22]. To avoid general contaminants and degradation products, reads shorter than 18 nt and longer than 31 nt were not included. The reference sequences were retrieved from NCBI, with accession numbers NC_003924.1 (CrPV), NC_015524.1 (MLV), NC_004146.1 (FHV, RNA1), NC_004144.1 (FHV, RNA2), NC_012958.1 *Drosophila* A virus (DAV), and NC_001834.1 *Drosophila* C virus (DCV). Plots depicting the length distribution and nucleotide frequency at each position were obtained in R statistical computing software (version 3.4.2) [23] with the viRome package (version 0.10) [24].

## 3. Results

### 3.1. Generation of CrPV-Derived siRNAs with Species-Dependent Length Distribution

**Figure 1:**
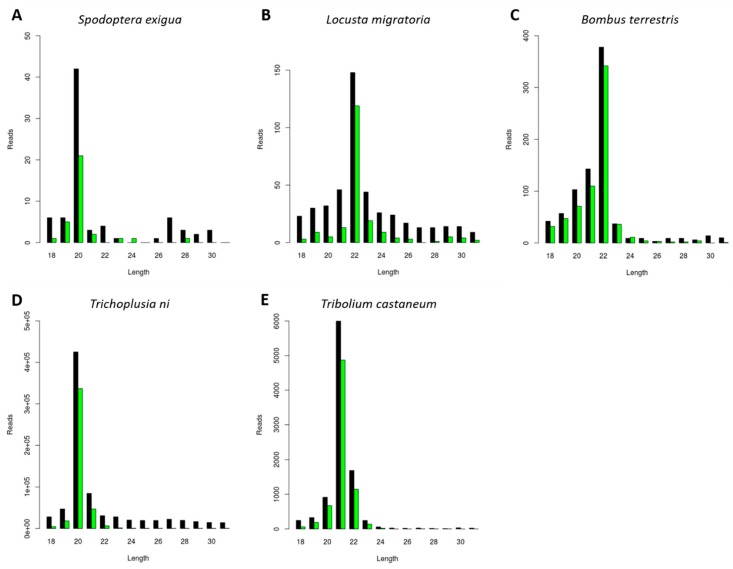
Generation of Cricket Paralysis Virus (CrPV)-derived small interfering RNAs (siRNAs) with species-dependent length distribution. Length distribution of small RNAs mapping to CrPV, derived from fat body of CrPV-infected *Spodoptera exigua* (**A**), *Locusta migratoria* (**B**), and *Bombus terrestris* (**C**); as well as from CrPV-infected *Trichoplusia ni* High Five cells (**D**) and *Tribolium castaneum* TcA cells (**E**). Reads (*y* axis): number of reads. Length (*x* axis): length (nt) of the reads. Black: sense reads. Green: antisense reads. To avoid degradation products and general contaminants, reads shorter than 18 nt and longer than 31 nt were not included. Mapping was performed with Bowtie2 and graphs were obtained with the viRome package in R.

In order to investigate the generation of CrPV-specific siRNAs, a CrPV infection was induced in five distinct insect species belonging to economically relevant orders. Specifically, the pest species *Locusta migratoria* (Orthoptera), *Tribolium castaneum* (Coleoptera), *Trichoplusia ni*, and *Spodoptera exigua* (Lepidoptera) were selected, as well as the beneficial insect *Bombus terrestris* (Hymenoptera). In each case, an in vivo or an in vitro approach was used: for *L. migratoria*, *S. exigua*, and *B. terrestris*, the fat body from CrPV-infected animals was used; in the case of *Trichoplusia ni* and *Tribolium castaneum*, the High Five and TcA cell lines were used, respectively. The small (s)RNAs were purified, sequenced and the length distribution of the reads mapping to CrPV was analyzed (Figure 1). For the investigated species, a sRNA length distribution with peaks ranging from 20 to 22 nt was observed (Figure 1), corresponding to the typical size of siRNAs. Interestingly, the majority of viral siRNAs from the lepidopteran species *S. exigua* and *Trichoplusia ni* were 20 nt in length (Figure 1A,D). For *L. migratoria* and B*. terrestris*, this peak appeared at 22 nt (Figure 1B,C), while in *Tribolium castaneum*, 21 nt appeared to be the favored siRNA length (Figure 1E).

### 3.2. Generation of FHV-Derived siRNAs with Species-Dependent Length Distribution

**Figure 2:**
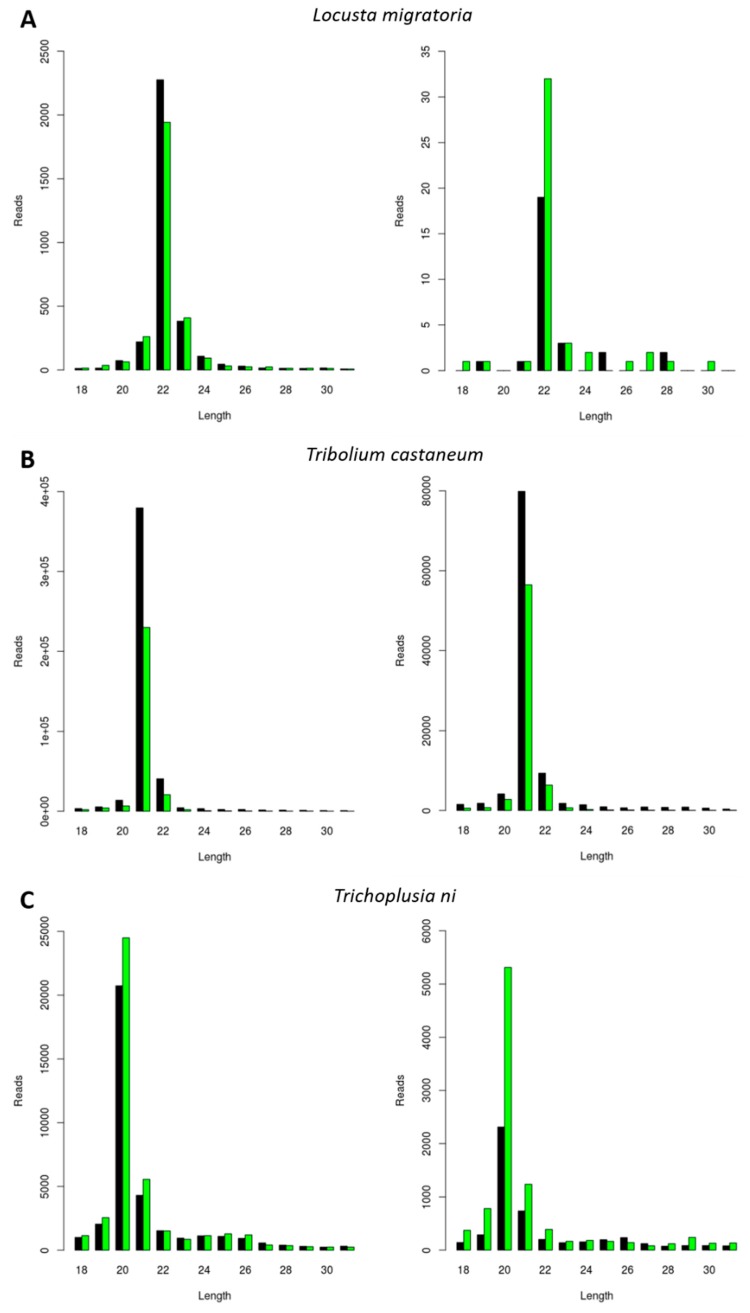
Generation of Flock House Virus (FHV)-derived siRNAs with species-dependent length distribution. Length distribution of small RNAs mapping to FHV, derived from fat body of FHV-infected *L. migratoria* (**A**), as well as from FHV-infected *Tribolium castaneum* TcA cells (**B**) and from persistently FHV-infected *Trichoplusia ni* High Five cells (**C**). Left: FHV RNA1. Right: FHV RNA2. Reads (*y* axis): number of reads. Length (*x* axis): length (nt) of the reads. Black: sense reads. Green: antisense reads. To avoid degradation products and general contaminants, reads shorter than 18 nt and longer than 31 nt were not included. Mapping was performed with Bowtie2, and graphs were obtained with the viRome package in R.

Next, we investigated the generation of FHV-derived siRNAs. The selection of the three experimental species was made based on the results obtained for the CrPV infection (as described in the previous paragraph). To this extent, *L. migratoria*, *Tribolium castaneum*, and *Trichoplusia ni* were selected based on their distinct siRNA length distributions, respectively peaking at 22, 21, and 20 nt (Figure 1). Consequently, the length of siRNAs was then analyzed in these species during FHV infection. Besides constituting a well-known model virus with the potential for practical applications in pest control, this virus was known to be persistently present in the *Trichoplusia ni* High Five cells stock available in our lab. We observed that the length of the sRNAs mapping to both RNA1 and RNA2 of FHV was typical of siRNAs (20 to 22 nt) and that their length distribution peaks were similar to the ones observed for CrPV-derived siRNAs, namely 22 nt in *L. migratoria*, 21 nt in *Tribolium castaneum*, and 20 nt in *Trichoplusia ni* (Figure 2).

### 3.3. Generation of Other Virally Derived siRNAs with Species-Dependent Length Distribution

**Figure 3:**
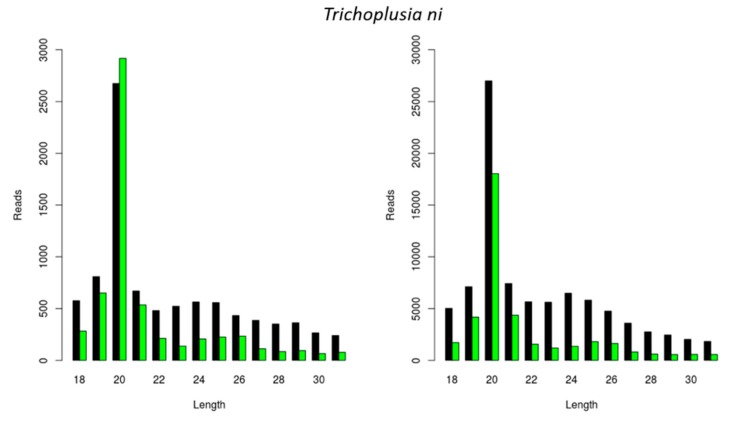
Generation of Macula-like Latent Virus (MLV)-derived siRNAs in two stocks of *Trichoplusia ni* High Five cells. Length distribution of small RNAs mapping to the MLV in a stock of High Five cells persistently infected with MLV and induced with a CrPV infection (**left**); and in a stock of High Five cells persistently infected with MLV and the FHV (**right**). Reads (*y* axis): number of reads. Length (*x* axis): length (nt) of the reads. Black: sense reads. Green: antisense reads. To avoid degradation products and general contaminants, reads shorter than 18 nt and longer than 31 nt were not included. Mapping was performed with Bowtie2, and graphs were obtained with the viRome package in R.

We further investigated if the previously observed species-dependent length distribution of virally derived siRNAs was restricted to CrPV and FHV (Figure 1 and Figure 2) or whether a similar length distribution would be observed for other viral infections. For this, we analyzed the length of siRNAs originating from a Macula-like Latent Virus (MLV) infection in *Trichoplusia ni* High Five cells. For this, we made use of two already available sRNA sequencing libraries: one originating from High Five cells persistently infected with MLV and in which a CrPV infection was induced (used in Figure 1D), and the other originating from High Five cells persistently infected with MLV and the FHV (used in Figure 2C). In both cases, the length distribution peak of MLV-derived sRNAs was 20 nt (Figure 3).

**Figure 4:**
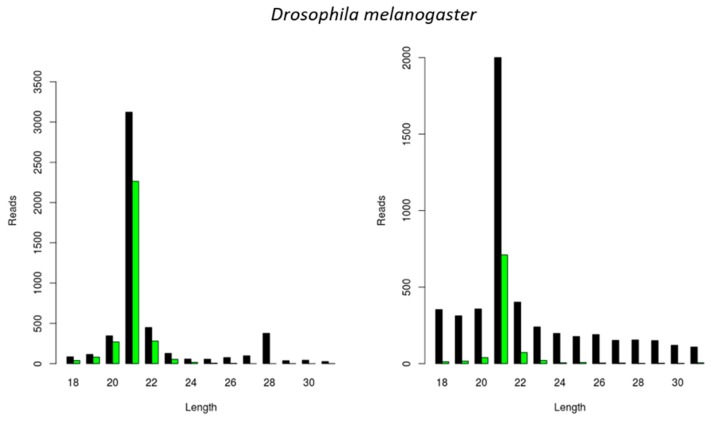
Generation of virus-derived siRNAs in *Drosophila melanogaster* S2 cells. Length distribution of small RNAs mapping to the *Drosophila* A virus (DAV) (**left**) and to the *Drosophila C* virus (DCV) (**right**). Reads (*y* axis): number of reads. Length (*x* axis): length (nt) of the reads. Black: sense reads. Green: antisense reads. To avoid degradation products and general contaminants, reads shorter than 18 nt and longer than 31 nt were not included. Mapping was performed with Bowtie2, and graphs were obtained with the viRome package in R.

In the following step, we investigated the situation in the well-known model organism *D. melanogaster*. For this, we used an S2 cell line stock available in our lab and started by identifying persistently infecting viruses, as previously described [17,18]. This resulted in the identification of two viral genomes that have been previously reported to maintain persistent infections in *D. melanogaster*, namely the *Drosophila* A Virus (DAV) and the *Drosophila* C Virus (DCV). We then analyzed the length of the siRNAs mapping to these viral genomes and verified that, for both viruses, the length distribution peak was at 21 nt (Figure 4).

### 3.4. Generation of dsRNA-Derived siRNAs with Species-Dependent Length Distribution

**Figure 5:**
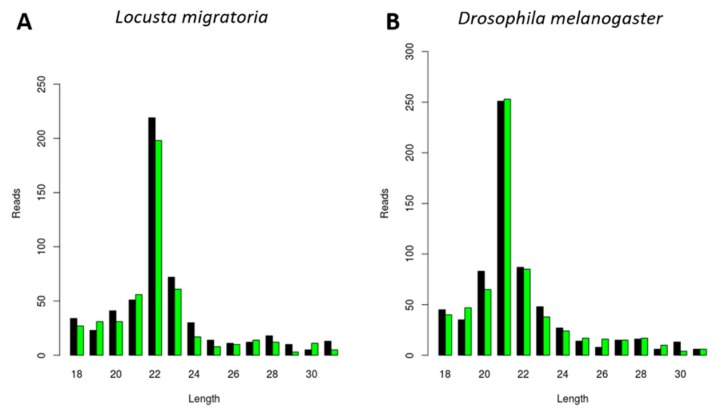
Generation of *luciferase* dsRNA (ds*luc*)-derived siRNAs with species-dependent length distribution. Length distribution of small RNAs mapping to the sequence of *luciferase* dsRNA, derived from midgut of ds*luc*-injected *L. migratoria* (**A**) and derived from *D. melanogaster* S2 cells treated with ds*luc* (**B**). Reads (*y* axis): number of reads. Length (*x* axis): length (nt) of the reads. Black: sense reads. Green: antisense reads. To avoid degradation products and general contaminants, reads shorter than 18 nt and longer than 31 nt were not included. Mapping was performed with Bowtie2, and graphs were obtained with the viRome package in R.

DsRNA molecules are important PAMPs (pathogen-associated molecular patterns) for viral infections by triggering the antiviral RNAi response. In addition, in-field delivery of dsRNA promises to contribute to RNAi-based insect pest control strategies. Therefore, we next studied if the length of dsRNA-derived siRNAs was also species-dependent, as well as whether it correlated with the length of virally derived siRNAs. For this, we analyzed the length of siRNAs derived from *luciferase* dsRNA (ds*luc*) in *L. migratoria* upon in vivo ds*luc* injection, as well as in *D. melanogaster* S2 cells upon in vitro ds*luc* delivery. The siRNA length varied from 20 to 22 nt, as expected for siRNAs (Figure 5). Interestingly, the ds*luc*-siRNA length was species-dependent, with length distributions peaks at 22 nt and 21 nt in *L. migratoria* and *D. melanogaster*, respectively (Figure 5), corresponding to all previous results.

## 4. Discussion

RNAi is a world-renowned technology with important applications in several fields, such as in the study of knockdown phenotypes in functional genomics research or for the development of novel, highly specific insecticides in pest control [25].

Insect Dicer2 recognizes dsRNA molecules and dices them into small interfering RNAs (siRNAs), the key effectors of the RNAi silencing mechanism. Dicer proteins typically comprise six main functional domains, namely a helicase domain (DExD/H, TRBP-BD, and HELICc), a DUF283, a PAZ (Piwi/Argonaute/Zwille) domain, two RNase III domains, and a dsRNA-binding domain [26]. Although insect siRNAs are generally described to be 18 to 24 nt in length, research has mainly focused on the model organism *D. melanogaster*. In this species, siRNAs are mostly 21 nt long, and their structure and length have been demonstrated to affect RNA silencing efficiency [2,3,4]. In this article, we describe a species-dependent siRNA length. Specifically, virus-derived siRNAs in the investigated lepidopteran species mostly exhibited a length of 20 nt, as was demonstrated in the *S. exigua* fat body and in *Trichoplusia ni* High Five cells (Figure 1A,D, Figure 2C and Figure 3). This was in accordance with what has been previously demonstrated for *Bombyx mori*, both in BmN4 cells and in vivo [18,27,28]. In *Tribolium castaneum*, we report 21-nt-long siRNAs for two distinct viruses (Figure 1E and Figure 2B). Although *Tribolium castaneum* is an important model organism to investigate the RNAi mechanism, no other siRNA size distribution was found in the literature, nor for any other beetle species. In *B. terrestris*, the size distribution of fat body virus-derived siRNAs presented a peak at 22 nt (Figure 1C). In line with this, 22-nt-long siRNAs have been described for several viruses in the honey bee, *Apis mellifera* [29]. Regarding *L. migratoria*, siRNAs were found to mostly be 22 nt long. This was demonstrated here for virus-derived siRNAs from the fat body, as well as for dsRNA-derived siRNAs from the midgut (Figure 1B, Figure 2A and Figure 5A). Although other reports of locust siRNAs were not found in literature, sRNA libraries of solitarious and gregarious locusts have shown a clear length distribution peak at 22 nt [30]. In addition, we report that the length of virus- and dsRNA-derived siRNAs in *D. melanogaster* S2 cells was mainly 21 nt (Figure 4 and Figure 5B), which is in agreement with previous reports [17,31,32,33]. Moreover, this is also the length of siRNAs in *Aedes* and *Culex* mosquitoes [34,35,36,37]. Taken together, and considering the current available literature, our data suggests that the length distribution of virus- and dsRNA-derived siRNAs is insect species-dependent. While the factors determining siRNA length in insects remain to be unraveled, studies performed with human Dicer have proposed that the length of small RNAs is determined by the interactions between the PAZ domain and the catalytic center formed by the two RNase III domains of this enzyme [38,39,40,41]. Interestingly, insect RNAi genes such as *dicer2* and *argonaute2* are described to be fast evolving, likely due to their role in antiviral immunity [42,43,44]. Consequently, it is interesting to observe that more closely related species seem to exhibit similar siRNA length patterns (*e.g.*, within the same order, as discussed above for lepidopteran, hymenopteran or dipteran species). This is possibly determined at the Dicer2 cleaving level and reinforced at the Argonaute2 binding selectivity level. Nevertheless, in order to confirm this hypothesis, further research must be performed, including comparative studies on the structure of Dicer and Argonaute proteins. In addition, evolutionary studies of insect and other arthropod Dicer proteins would be of great interest to further understand species-related RNAi variabilities.

The use of RNAi technology in insects still holds limitations in several species, specifically regarding the efficient delivery of dsRNA in the field. In this regard, viruses have been seen as possible candidates for dsRNA delivery systems [7,9]. These agents combine the capacity of entering insect cells with the ability of delivering/originating RNAi-triggering dsRNA molecules in insect cytoplasm. Due to their small size, simple genomic organization and well-described replication cycles, small RNA viruses such as FHV and CrPV constitute good candidates for dsRNA delivery. In fact, a FHV engineered to trigger RNAi in *D. melanogaster*, a species known to be generally refractory to RNAi technology, has been successfully designed [10]. This is an important proof of concept and encourages further research on these matters. In the current paper, we demonstrate that FHV successfully induces the creation of siRNA, the main RNAi effectors, in the fat body of *L. migratoria* in vivo, as well as in *Tribolium castaneum* and *Trichoplusia ni* cell lines (Figure 2). In addition, we report that CrPV-specific siRNAs were also formed in the fat body of *L. migratoria* and *S. exigua*, as well as in *Trichoplusia ni* and *Tribolium castaneum* cell lines (Figure 1). Thus, we propose the use of FHV and/or CrPV in future research for the development of viral-based RNAi-triggering systems in these four pest species belonging to three economically important insect orders. Keeping in mind a pest control purpose, it is of note that the results obtained in cell lines might not reflect the situation in vivo. In addition, the in vivo viral infections were obtained upon injection of the viral suspension, instead of via feeding. Therefore, although these are promising results, further research on these matters is needed. Notably, CrPV was demonstrated to also induce the generation of siRNAs in the beneficial species *B. terrestris*, the bumblebee (Figure 1C). Therefore, possibilities of obtaining species specificity must be taken into high consideration in future studies as well. Importantly, since RNAi acts at the nucleotide sequence level, pest control specificity can be achieved via dsRNA sequence selection. For instance, by carefully selecting the dsRNA sequence region, species-specific knockdown has already been achieved in four closely related *Drosophila* species [45].

Another noteworthy feature of insect RNAi is that, in opposition to exposure to longer dsRNA molecules, direct exposure to short dsRNA molecules, such as siRNAs, does not generally result in an RNAi response. Nevertheless, some exceptions have been reported [46,47]. In this regard, the species-dependent siRNA length here reported might contribute to an improved design of synthetic siRNAs, depending on the target species.

Studies have demonstrated that certain viruses have the potential to interfere with the outcome of other viral infections [48,49,50]. In this respect, it is interesting to observe that, although the MLV was persistently present in High Five cells, a CrPV infection still induced the generation of siRNAs (Figure 1D). Similarly, a stock of High Five cells that was persistently infected by MLV and FHV revealed the generation of siRNAs for both viruses (Figure 2C and Figure 3). In line with this, the DAV and DCV were found to be persistently infecting our stock of *D. melanogaster* S2 cells, and siRNAs of both viruses were present (Figure 4). In addition, these cells generated dsRNA-specific siRNAs in the presence of ds*luc* (Figure 5B). Currently, it is known that persistent viruses are often present not only in stocks of insect cell lines, but also in insect populations [17,18,51,52,53,54,55,56,57,58,59]. Therefore, the mentioned observations are particularly relevant when considering using RNAi and viral-based RNAi systems for pest control.

## 5. Conclusions

In this study, we report the generation of FHV- and/or CrPV-derived siRNAs in five insect species. In addition, we demonstrate a species-dependent siRNA length upon dsRNA delivery and/or viral infection in six different insect species belonging to five economically important insect orders (Lepidoptera, Orthoptera, Hymenoptera, Coleoptera, and Diptera). These results have implications for several applied insect research fields, namely regarding RNAi-based pest control in general, as well as viral-based RNAi-triggering systems.
